# Harnessing the Immune System with Cancer Vaccines: From Prevention to Therapeutics

**DOI:** 10.3390/vaccines10050816

**Published:** 2022-05-21

**Authors:** Ilene Le, Subramanian Dhandayuthapani, Jessica Chacon, Anna M. Eiring, Shrikanth S. Gadad

**Affiliations:** 1 Paul L. Foster School of Medicine, Texas Tech University Health Sciences Center El Paso, El Paso, TX 79905, USA; ilene.le@ttuhsc.edu (I.L.); s.dhandayuthapani@ttuhsc.edu (S.D.); jessica.chacon@ttuhsc.edu (J.C.); 2 L. Frederick Francis Graduate School of Biomedical Sciences, Texas Tech University Health Sciences Center El Paso, El Paso, TX 79905, USA; 3 Center of Emphasis in Infectious Diseases, Department of Molecular and Translational Medicine, Texas Tech University Health Sciences Center El Paso, El Paso, TX 79905, USA; 4 Center of Emphasis in Cancer, Department of Molecular and Translational Medicine, Texas Tech University Health Sciences Center El Paso, El Paso, TX 79905, USA; 5 Mays Cancer Center, UT Health San Antonio MD Anderson Cancer Center, San Antonio, TX 78229, USA

**Keywords:** cancer, cancer vaccines, antigens, immunotherapy

## Abstract

Prophylactic vaccination against infectious diseases is one of the most successful public health measures of our lifetime. More recently, therapeutic vaccination against established diseases such as cancer has proven to be more challenging. In the host, cancer cells evade immunologic regulation by multiple means, including altering the antigens expressed on their cell surface or recruiting inflammatory cells that repress immune surveillance. Nevertheless, recent clinical data suggest that two classes of antigens show efficacy for the development of anticancer vaccines: tumor-associated antigens and neoantigens. In addition, many different vaccines derived from antigens based on cellular, peptide/protein, and genomic components are in development to establish their efficacy in cancer therapy. Some vaccines have shown promising results, which may lead to favorable outcomes when combined with standard therapeutic approaches. This review provides an overview of the innate and adaptive immune systems, their interactions with cancer cells, and the development of various different vaccines for use in anticancer therapeutics.

## 1. Introduction

Prior to the development of smallpox vaccination by Edward Jenner in the eighteenth century [1], immunization and protection methods against infectious diseases provided unpredictable results for patients [2]. Since its discovery, many scientific pioneers have fine-tuned the techniques necessary for vaccine development, which paved the way for modern vaccination protocols [2,3]. The protective nature of vaccines has resulted in the prevention of infections and the eradication of many different diseases. For example, some diseases that are now preventable through immunization include tetanus, diphtheria, tuberculosis, influenza, measles, mumps, rubella, hepatitis, and varicella-zoster, among others [2,4]. Interestingly, immunization against some viral infections, such as the human papillomavirus or hepatitis, can also prevent the development of cervical and liver cancer, respectively, by preventing infection with cancer-causing viruses [5,6]. In more recent years, studies have evaluated whether vaccines can also be used in cancer therapy [7,8]. With the success of vaccines in containing infections utilizing the host’s immune system, research is now focused on developing methods to harness this technology for cancer prevention and elimination [9]. However, progress has been slow due to the lack of validated biomarkers that predict vaccine efficacy, challenges relating to vaccine stability and delivery, and the costs associated with the production of personalized patient-specific vaccines [10]. As vaccines move from disease prevention to therapy, cancer vaccines are becoming an integral part of therapeutic strategies for tertiary and primary cancer prevention [11].

**Figure 1:**
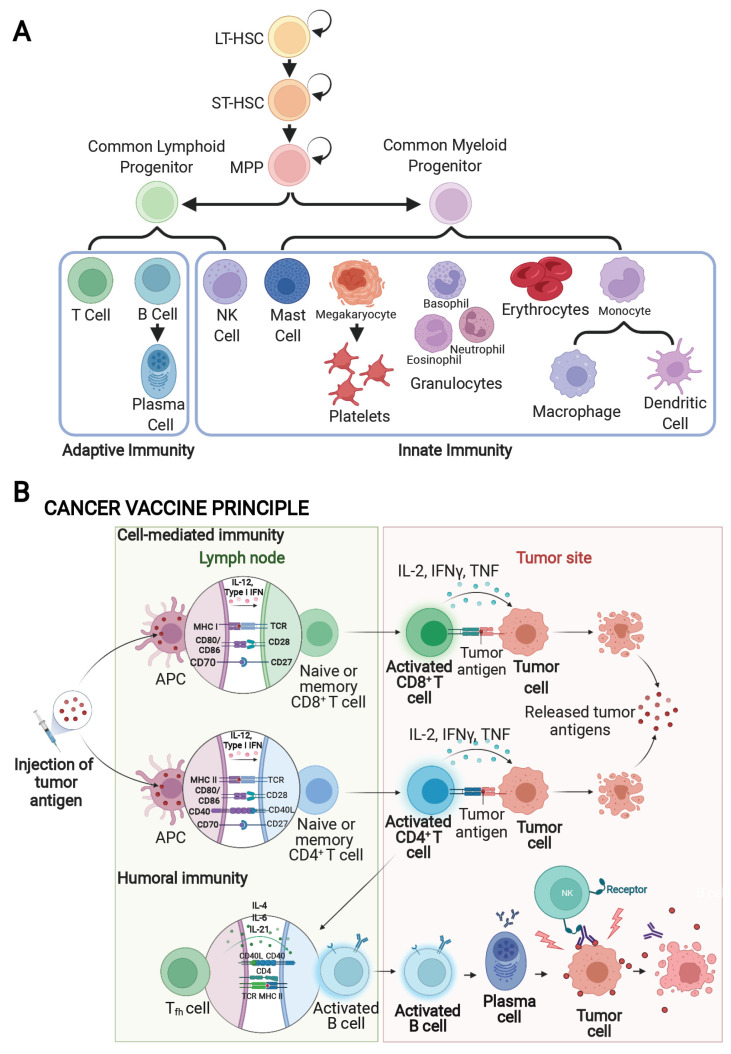
Innate versus adaptive immunity and the mechanisms by which cancer vaccines activate the immune system. (**A**) The schematic shows the hierarchy of hematopoietic lineage commitment, divided into innate versus adaptive immunity. Created using “Immune & Blood Cells”, by BioRender.com (accessed on 26 January 2022). Retrieved from https://app.biorender.com/categories/cell-types/ (accessed on 26 January 2022). LT-HSC, long-term hematopoietic stem cell; MPP, multipotent progenitor; NK cell, natural killer cell; ST-HSC, short-term hematopoietic stem cell. (**B**) The schematic shows multiple mechanisms by which cancer vaccines activate the immune system, both in the tumor site and within the lymphatic system, divided into cell-mediated versus humoral immunity. Adapted from “Cancer Vaccine Principle” by BioRender.com (accessed on 26 January 2022). Retrieved from https://app.biorender.com/biorender-templates (accessed on 26 January 2022). APC, antigen-presenting cell; CD, cluster of differentiation; IFN, interferon; IL, interleukin; MHC I, major histocompatibility complex I; MHC II, major histocompatibility complex II; T_fh_, CD4^+^ T follicular helper cells; TNF, tumor necrosis factor.

The current standard of care for cancer treatment consists of various options, including surgery, chemotherapy, radiotherapy, hormonal therapy, molecularly targeted therapy, and immunotherapy, which provide variable results due to several factors [12]. Immunotherapy focuses on harnessing the host immune system, both humoral and cellular, to attack malignant cells [13]. The immune system is a complex network of cells and proteins that provide innate (general) and adaptive (specific) defense mechanisms for the body (Figure 1A). Innate immunity includes anatomical barriers and physiological barriers (e.g., skin, mucous membranes), endocytic and phagocytic barriers (e.g., macrophages, neutrophils, natural killer cells), and inflammatory barriers (e.g., complement) [14,15]. The phagocytic macrophages of the innate immune system generally provide the first line of defense against many different microorganisms and are essential for controlling common bacterial infections. In addition to cellular immunity, the innate immune system also consists of proteins of the complement system, which can form pores directly in the bacterial cell surface, thereby killing the pathogen [16]. Notably, the innate immune response makes a crucial contribution to the activation of the adaptive immune system. Adaptive immunity functions to differentiate self-antigens from non-self-antigens, eliminate the pathogen or the infected cells, and produce immunologic memory in case there is a future infection with the same pathogen [15,17].

Adaptive immunity is also responsible for clearing the body of cancerous cells. Instead of bearing several different receptors such as the cells of the innate immune system, lymphocytes of the adaptive immune system bear antigen receptors of a single specificity. While each lymphocyte carries receptors targeting only one antigen, each is different, providing millions of diverse antigen receptor specificities. There are two branches of immunity within the adaptive immune system: antibody-mediated (humoral) immunity from B cells and cell-mediated (cellular) immunity from T cells [18]. While both components are essential, the cell-mediated mechanisms play a more prominent role in cancer clearance due to the killing capabilities of CD8^+^ cytotoxic T lymphocytes (CTLs) [18]. The methods employed by cell-mediated immunity include apoptosis of cells displaying foreign antigens, activation of macrophages and natural killer cells to destroy pathogens, and potentiating the immune response by stimulating cytokine production [15]. All the intricacies of the immune system work together to protect and remove any foreign material from the body [18].

Similarly, with the utilization of cancer vaccines, the host immune system can be redirected to target cancerous cells (Figure 1B) that find ways to evade the immune response [19]. Tumor antigens for the development of cancer vaccines can originate from genetic components such as DNA and mRNA, purified tumor proteins, long synthetic peptides, and tumor lysates [20]. Methods for antigen delivery include viral-based delivery, nanoparticles, and dendritic cell delivery [20,21,22,23,24]. The Food and Drug Administration (FDA) is now beginning to approve cancer vaccines as their development and efficacy are confirmed, such as the recently approved Sipuleucel-T (PROVENGE; Dendreon) for the treatment of recurrent prostate cancer [25]. The goal of cancer vaccination strategies is to induce antigen-specific, B cell-based humoral immunity and T cell-based cellular immunity that are capable of targeting and clearing the cancerous cells and inducing long-term immunological memory (Figure 1B). However, this becomes problematic when cancer cells evade the immune system, and strategies to overcome this problem are currently being employed in cancer therapeutics.

Vaccines regulate or modulate anti-tumor immune responses; for example, administration of Sipuleucel-T leads to elevation of antigen-specific T cells, and activated lymphocytes are directed against tumors [26]. Notably, Sipuleucel-T stimulated a humoral immune response to other tumor antigens, triggering an anti-tumor cascade and improving clinical outcomes [27]. On the other hand, Talimogene laherparepvec (T-VEC), another oncolytic viral vaccine approved for melanoma therapy, selectively lyses the tumor cells to release tumor antigens [28,29] and also secretes granulocyte-macrophage colony-stimulating factor (GM-CSF), thereby recruiting dendritic cells (DC) to the tumor [30].

In this review, we will discuss the common interactions between cancer cells and the immune system, harnessing the immune system for cancer therapy, and the current state of vaccines for use in cancer prevention and treatment.

## 2. Evasion of the Immune System by Cancerous Cells

For the adaptive immune system to mount an efficient anticancer response, a series of events must be initiated and allowed to proceed, known as the Cancer-Immunity Cycle [31]. The first step involves the production of cancer-specific antigens, known as neoantigens, that are released and captured by dendritic cells (DCs) for processing. To induce an anticancer response, the presentation of these antigens must be accompanied by signals (e.g., cytokines) that specify tumor immunity versus tolerance. In the next step, DCs present the captured antigens associated with major histocompatibility complex I (MHC class I) or II (MCH class II) on the surface of their cell. In the presence of the proper costimulatory molecules, engagement of the T cell receptor with MHC:antigen complexes on DC cells results in priming and activation of effector T cell responses. Finally, the activated T cells migrate and infiltrate the tumor, which presents the processed neoantigens in complex with MHC class I on the cell surface, resulting in cancer cell killing. Dying cancer cells release further tumor-associated antigens, thereby potentiating the process [31].

Each step of the Cancer-Immunity Cycle is coordinated by many different factors, including molecules that are either stimulatory or inhibitory. Stimulatory factors promote immunity, whereas inhibitory molecules keep the process in check to prevent autoimmunity [31]. Unfortunately, cancer cells can evade the immune response in several different ways. For example, tumor antigens may not be detected, DCs and T cells may develop tolerance to the antigen, treating it as self rather than foreign, or T cells may not properly home to the tumor site. Additionally, T cells can be prevented from infiltrating the tumor upon arrival, or factors present within the tumor microenvironment may suppress the effector T cell function [31,32].

The first mechanism used by cancer cells to suppress the immune response is by downregulating MHC class I, which is required for lymphocyte activation when complexed with a foreign antigen. Additionally, some tumor cells will downregulate the costimulatory molecules that are necessary for full T cell activation (see Figure 1B) [33]. Either mechanism results in a loss of the antigen presentation machinery, allowing the cancer cells/antigens to remain undetected by the immune system [34]. Cancer immune evasion can also be achieved through the binding of programmed death ligand-1 (PD-L1) or -2 (PD-L2) on the cancer cells to programmed cell death protein-1 (PD-1) on the surface of T cells, which inhibits T cell activation by inducing T cell exhaustion [35,36]. In a similar manner, CTL-associated antigen-4 (CTLA-4) on the surface of cancer cells can interact with CD80/CD86 costimulatory molecules on T cells, thereby blocking full T cell receptor activation by foreign antigen. Indeed, monoclonal antibodies that bind to PD-L1, PD-1, or CTLA-4, known as immune checkpoint inhibitors (ICIs), are now being used for the treatment of multiple different human malignancies, which ultimately turn on the patient’s immune system to target their specific type of cancer [36].

While the adaptive immune system can perform immunosurveillance to prevent cancer development, innate immunity and the process of inflammation can promote tumorigenesis [37]. Indeed, tumor-associated inflammation can, in some cases, lead to alterations that drive tumorigenesis and disease progression. Notably, the presence of intratumoral cancer-associated fibroblasts (CAFs), macrophages, myeloid-derived suppressor cells (MDSCs), and T regulatory cells (Tregs) can act as key sources of immune-inhibitory factors within the tumor microenvironment [31]. CAFs play a prominent role in supporting the growth of tumor cells, remodeling the extracellular matrix, promoting angiogenesis, and promoting inflammation [38]. In addition, CAFs regulate various cancer-related phenotypic outcomes such as extracellular matrix (ECM) remodeling, induction of pro-cancer growth molecules, and interaction with drug or other therapy-based regimens [20]. Recent studies have shown the role of CAFs in modulating the immune response, and efforts are currently exploring CAFs as a therapeutic target in cancer treatment. However, CAF-based therapeutic strategies may have significant challenges due to their involvement in pro- and anti-tumor responses [20].

Macrophages within the tumor microenvironment can exist in a pro-tumor phenotype (M2-like) or an anti-tumor phenotype (M1-like) [39]. The M2-like phenotype can promote tumorigenesis and metastasis by secreting cytokines and growth factors and promoting the expression of inhibitory molecules such as PD-1 [39,40]. A method to evade T cell-mediated killing includes upregulating immune checkpoints, which represses the activation of T cells [41,42]. Targeting those immunosuppressive cytokines can lead to T cell reactivation and tumor clearance [43]. Additionally, macrophages can help recruit Tregs and MDSCs to the tumor microenvironment, where they exhibit potent immunosuppressive activity [32,44]. Altogether, the ability of cancer cells to evade recognition by the immune system in part explains the reduced response to chemotherapy observed in certain cancers and cancer patients. All of these mechanisms of cancer immune evasion are currently being studied as novel targets in cancer therapy [32].

## 3. Cancer Immunotherapy

Cancerous cells can reside in the host body undetected through a variety of different regulatory processes, as described above [45]. Immunotherapy is a new form of cancer therapy focused on harnessing the host immune system to attack specific types of cancer cells [13]. Immunotherapy exists in both passive and active forms, such as adoptive cellular immunotherapy, natural killer cell therapy, chimeric antigen receptor T (CAR T) cell therapy, and the use of ICIs [46]. Adoptive T cell therapy allows for in vitro growth of patient-derived tumor antigen-specific T cells that are then reintroduced back into the patient [33]. Since Tregs are suppressive in the tumor microenvironment, lymphodepletion approaches are performed prior to re-infusing the T cell product back into the patient [43,47]. Adoptive cell therapy relies on the immune system to recognize tumor cells by modifying tumor-infiltrating lymphocytes, T cell receptors, or introducing chimeric antigen receptors [48]. When combined with cancer vaccines, adoptive cell therapy was shown to provide synergetic effects in solid skin tumors [49]. On the other hand, natural killer (NK) cell therapy focuses on the cells’ innate ability to recognize and eliminate cancerous cells without prior sensitization [50]. In metastatic solid tumors, clinical trials have demonstrated that activation of NK cells provides better immunotherapy outcomes when compared with T cells [51]. Immune checkpoint proteins, such as CTLA-4 and PD-1, prevent T cells from destroying cancer cells, as described above [41]. The PD1-Vaxx vaccine (Imugene Ltd., Sydney, Australia) produces polyclonal antibodies that inhibit PD-1 in breast and pancreatic cancer cells [52], resulting in a significant decrease in tumor growth in mice [52]. As a consequence, Imugene Ltd. has received FDA approval for clinical testing.

Adoptive T cell transfer, ICIs, and bispecific antibodies are the most prevalent types of immunotherapies [53]. Although ICI-based immunotherapy has shown remarkable progress in cancer treatment, many cancers relapse over time [54]. However, due to this relapse, research is now focused on developing combinatorial therapies, including ICIs and cancer vaccines [55]. Cancer vaccines, as compared with ICIs, have the advantage of utilizing the entirety of the host immune system, instead of just an individualized component, for cancer cell targeting [56]. As demonstrated in preclinical models, when both are combined, treatment success is greatly improved [20,29]. This makes cancer vaccines an area of interest to pursue further.

## 4. Cancer Vaccines

**Figure 2:**
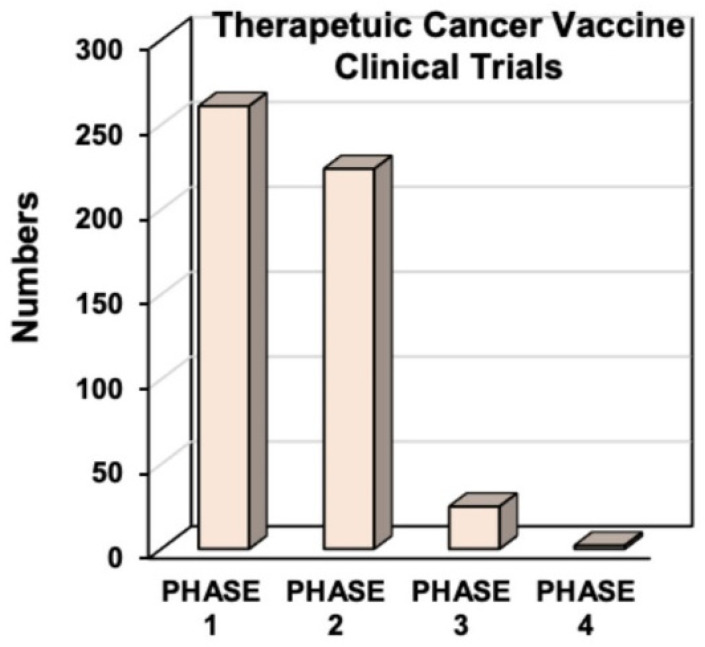
Cancer vaccines in clinical trials. The bar graph shows the frequency of therapeutic cancer vaccines in the USA, divided by phase of the clinical trial. Data were extracted from www.clinicaltrials.gov (accessed on 14 June 2021). Created with https://BioRender.com (accessed on 26 January 2022).

Similar to the mechanism of action for immunotherapy, cancer vaccines also utilize the host immune system to treat cancer. Cancer vaccines can elicit a cancer-specific immune response and diminish tumor size in patients [56]. Current cancer vaccines employ the activation of either humoral or cellular adaptive immune responses. The humoral approach generates antibodies based on tumor antigens presented on intact cancer cells (Figure 1B) [57]. For example, Sipuleucel-T (Provenge), a dendritic cell vaccine (discussed below) that is approved for use in some men with metastatic prostate cancer, stimulates an immune response to prostatic acid phosphatase, an antigen present in most prostate cancers [58]. These vaccines increase the level of IgG antibodies targeting tumor-specific antigens, thereby promoting the priming of T cells and their ability to detect cancer [59]. In the cellular process, T cells directly mount an immune response against protein-based tumor antigens (Figure 1B) [57]. The Sipuleucel-T vaccine showed a small but significant increase in survival of prostate cancer patients by about four months [60]. The cellular approach allows a broader immunologic effect, and most cancer vaccines aim to induce T cell activation [61]. Sipuleucel-T is recommended to treat men with metastatic prostate cancer, both asymptomatic and castration-resistant. Currently, numerous cancer vaccines are undergoing different phases of clinical trials to assess their therapeutic utility (Figure 2).

### 4.1. Cancer Antigens

When discussing tumor antigens, they can be classified into two general categories: tumor-associated and tumor-specific. Tumor-associated antigens (TAAs) can be found in tumor tissue, but they can also be present in normal tissue [62,63]. Because of their natural expression in the host, these antigens are involved in central and peripheral tolerance, leading to a weaker response due to depletion of high-affinity TAA-specific T cell receptors [64,65]. One of the first TAAs studied was carcinoembryonic antigen, which was found to be overexpressed in colorectal cancer [66]. Investigations demonstrated that the inadequate immune response was due to TAA expression on both cancerous and normal epithelial cells [66]. However, because of the decreased immune response due to tolerance and lack of specificity for the tumor, there are concerns about potential toxicity from increased dosing to provide a more potent effect [65,67].

Tumor-specific antigens, or neoantigens, are tumor antigens that are solely expressed by cancer cells and not in normal tissue. Because the neoantigens are generally not present on normal host cells such as TAAs, neoantigens do not generate central and peripheral tolerance, making them a better target for therapy [64]. It was previously shown that tumor antigens have a more robust individual specificity engaged in stronger rejection of the tumor [62]. This indicates that neoantigens, compared with TAAs, can elicit a stronger immune response [62]. Furthermore, mutations in tumor cells can alter the amino acid sequence of peptides, leading to the formation of neoantigens that can be used for cancer vaccine development.

For this reason, neoantigens are highly cancer-specific when compared with TAAs [67]. Neoantigens, such as Neu-glycolyl-GM3 ganglioside, are overexpressed in multiple solid tumors. The Racotumomab vaccine has been shown to mimic ganglioside and have fair outcomes in patients with non-small-cell lung cancer [68]. Greater specificity leads to the generation of more robust immune responses. Therefore, neoantigens could provide a better target for developing vaccines for cancer therapy [69]. However, limitations to this approach include the necessity of sufficient sequencing data to determine the neoantigens present in individual patients and the high cost of production [70].

### 4.2. Types of Cancer Vaccines

The antigens used for cancer vaccines are divided into three major types: cellular, peptide/protein, and genetic (Table 1) [64,71]. Cellular vaccines can be further divided into whole tumor cell vaccines and dendritic cell vaccines. Whole tumor cell vaccines employ cancer cells that have been killed [67]. The target does not have to be identified beforehand, and there is non-specificity in the targeting of cancer [55,64,67]. Dendritic cell vaccines, in contrast, use autologous patient-derived dendritic cells that are loaded with peptide antigens or transfected with antigen genes [67]. Previous studies indicated a small but significant increase in the survival of patients with acute myeloid leukemia utilizing this approach [67,72]. While this vaccination method provides essential findings, the complexity and production costs have prevented frequent use [67].

Peptide/protein vaccines can be composed of tumor-associated antigens, cancer germline antigens, virus-associated antigens, or tumor-specific antigens [83]. The mechanism behind peptide vaccines is to generate T cells that are TAA-specific to mount an immune response [84]. These vaccines are relatively stable and safe, but suffer from the limitation of epitopes for potential vaccine targets, weaker immunogenicity of tumor antigens, and immune evasion [64]. While peptide vaccines have the advantage of using synthetic peptides, the disadvantage is that the appropriate selection and modification of immunogens are necessary to elicit the desired immune response [84]. However, evidence has also shown that CD8^+^ T cells generated from protein-based vaccines are less effective than other vaccines [85].

Gene-based cancer vaccines utilize DNA and RNA to produce cancer-specific antigens from peptides and proteins to induce an immunologic response [64]. These vaccines act by delivering tumor antigen-encoding genes, thereby enhancing the immune response towards cells expressing those antigens [86]. Advantages of DNA vaccines include generating a systemic response and creating memory [86]. These vaccines can also deliver multiple genes simultaneously via the same delivery method [86]. An advantage of RNA vaccines over DNA vaccines is that transcription is unnecessary [20]. For this reason, they are further along in the process of antigen expression and MHC presentation. When using viral vectors or nucleic acids, the response of CD8^+^ T cells was shown to be effective and sustained [85]. However, there are still limitations to this vaccination method, including resistance due to tumor evolution, antigen tolerance, and an influx of inflammatory cells [86]. This vaccination method is also limited by the delivery method and the uptake efficiency into cells.

### 4.3. Approved Cancer Vaccines

While cancer vaccines are still being widely studied, many vaccines have been approved for cancer therapy (Table 1). After the FDA approval of Sipuleucel-T (Provenge) in 2010, exponential advances have been made in cancer vaccine development [57]. Sipuleucel-T is a dendritic cell vaccine used to treat prostate cancer based on modifying dendritic cells from the patient [27]. However, there is controversy as to whether this vaccination provides enough benefit to outweigh the costs [87]. Still, clinical studies have shown that this vaccine is safe and effective, at least to some degree [25,58,73]. In contrast, PSA-TRICOM (Prostvac-VF) is a recombinant viral vaccine used to treat prostate cancer [88], which was shown to improve survival rates by as much as eight months [73].

Some peptide-based vaccines are made from cancer-testis antigens, such as MAGE-A3 and NY-ESO1 [89,90]. These proteins are widely studied and can induce a humoral and cellular immune response in cancer patients. While further use of the vaccine was halted due to limited benefit to the patients, other studies were conducted to explore combination therapies, adjuvant selection, and patient selection criteria to improve efficacy [73,91,92]. Algenpantucel-L (HypeAcute Pancreas) is a whole-cell vaccine developed from human tumor cell lines [93]. This vaccine strategy covers human tumor cell lines with antigens that are lethally irradiated before being injected back into the host to induce an immune response [73].

Some vaccines are bacterial-based, for example, Bacille Calmette–Guérin (BCG), approved for use in the treatment of bladder cancer (1990). BCG is one of the most widely used vaccines globally, which can treat certain bladder infections and eliminate residual bladder cancer cells after surgical resection [94]. The mechanism of action for this vaccine likely employs a combination of its direct effect on tumor cells through internalization of BCG and activation of the innate immune system. BCG ultimately leads to bladder cancer cell death through intracellular signal pathway activation and the release of cytokines by the immune system [95,96,97].

Talimogene laherparepvec (T-VEC) is another example approved in 2015 by the FDA to treat lesions in recurrent melanoma [98]. T-VEC is derived from herpes simplex virus type 1, designed to replicate inside the tumors and release GM-CSF, resulting in tumor-specific immune responses. The GM-CSF gene in T-VEC was engineered to replace viral genes such as ICP34.5 and ICP47 [99]. T-VEC has been shown to improve durable response rates and overall survival (OS) in patients >18 years of age [98]. This genetic modification allows for an increased response from neutrophils while refocusing the target on malignant cells [98,100].

The current FDA-approved vaccines on the market for cancer therapy are the BCG vaccine, Sipuleucel-T vaccine, and Talimogene vaccine [101]. However, the most recent antigen-based cancer vaccine, PROSTVAC, with promising data in a phase II study [102], did not show any improvement in OS of castration-resistant prostate cancer (CRPC) in men aged 18 years or older [88]. The authors of the study suggest that the lack of an immune response or the inhibitory tumor microenvironment explains the failure of PROSTVAC in clinical trials. To enhance the efficacy of PROSTVAC, combination therapy involving ICIs is now being explored. There is a continual effort toward developing cancer vaccines that are safe and effective, which will be instrumental in the field of precision medicine.

### 4.4. Combination Therapies

While advances in cancer vaccine treatment have gained ground, cancer vaccines alone have not provided a strong enough response to eradicate cancer independently [57,103]. As cancer cells continue to evolve mechanisms avoiding immune system detection, it becomes necessary to invoke multiple methods for cancer eradication. Recent studies have shown that therapies combining previously studied drugs with cancer vaccines provide much more promising results [57,58]. Using combined techniques, the tumor’s initially impaired immune response could potentially be restored [104]. In addition, the efficacy of combination treatment has proven to be increased compared to that of monotherapy [104,105]. While vaccines can induce an immune reaction, solely using vaccines is not enough to elicit a sufficiently strong response to eradicate cancer [21]. Co-therapy of cancer vaccines with cytokines, radiotherapy, ICIs, small molecules, endocrine therapy, and/or chemotherapy have synergetic effects [57,86]. Combining previous methods of general cancer eradication with patient-specific treatments will provide better results and enhance overall survival [57,106,107,108]. While advances in cancer vaccines have made great strides, the direction for cancer eradication has moved towards combination therapies [29]. Previously, cancer vaccines were used as a last attempt. Still, the move to utilize them as part of the first-line treatment requires knowledge of when to administer for the appropriate immune response, the potential necessity for multiple doses, and the interaction between the therapies employed to provide the desired outcome [86,109].

## 5. Conclusions and Future Directions

Vaccinations have long protected humans from the devastating effects of infectious diseases and cancer. However, aspects of the innate and adaptive immune system are routinely utilized by cancer cells to evade immunologic responses in the host. The challenge is now to use vaccines as first-line cancer therapeutics. New vaccinations are being developed to target preexisting cancerous cells using the same techniques employed in cancer prevention. By targeting those mechanisms, cancer vaccines may also prevent cancer progression. Establishing and prioritizing immunogenic neoantigens will be critical to providing an optimal response during vaccine development. In addition, multiple different types of cancer vaccines can be employed to determine maximal effectiveness depending on the type of cancer. While research has shown promising results for cancer vaccines, additional studies have shown that the combination of cancer vaccines with previous standard therapies may provide the best results for cancer eradication.

There are still many challenges to overcome for vaccine-based anticancer therapeutics. Notably, the ability of T cells to respond to antigenic challenges is affected by numerous factors, including age, diet, gut microbiome, and the tumor microenvironment [110]. Potential areas of study for the future of cancer vaccines include tumors that are not responsive to immunotherapy [64]. Another issue is that a patient may express heterogeneity of tumor cells leading to inadequate treatment if the vaccine focuses on only one particular neoantigen [64]. This limitation could be mitigated by creating a vaccine targeting multiple neoantigens specific to the patient [111,112]. Indeed, BioNTech and Moderna are currently exploring the combination of several different patient-specific neoantigens in mRNA-based vaccine clinical trials in an attempt to realize personalized medicine in cancer therapy. However, producing an individualized, patient-specific vaccine is very expensive due to analysis and production costs [68,113]. While still limited in some aspects, the continued advancement in cancer vaccination will provide better treatment outcomes for patients in the future.

## Figures and Tables

**Table 1:** List of therapeutic vaccines.

Vaccine	Type of Vector	Type of Antigen	Cancer Type	References
Sipuleucel-T (Provenge)	Dendritic cell	Tumor-associated: Prostatic acid phosphatase	Prostate cancer	[73,74]
Bacille Calmette-Guérin (BCG)	Bacteria	Tumor-associated: Thomsen–Friedenreich (T) antigen and sialyl-T (sT)	Bladder Cancer	[75,76]
Talimogene laherparepvec (T-VEC)	Viral	Tumor-associated: US12	Melanoma	[77,78]
PSA-TRICOM (Prostvac-VF)	Viral	Tumor-associated: Prostate-specific antigen	Prostate cancer	[73,79]
MAGE-A3	Peptide	Neoantigen	Lung cancer Melanoma	[73,80]
NY-ESO1	Peptide	Cancer-Testis antigen	Esophageal squamous cell carcinoma	[73,81]
Algenpantuecel-L (HyperAcute Pancreas)	Whole-cell	Tumor-associated: αGal	Pancreatic adenocarcinoma	[73,82]
